# U−shaped association between the glycemic variability and prognosis in hemorrhagic stroke patients: a retrospective cohort study from the MIMIC-IV database

**DOI:** 10.3389/fendo.2025.1546164

**Published:** 2025-04-03

**Authors:** Yuchen Liu, Houxin Fu, Yue Wang, Jingxuan Sun, Rongting Zhang, Yi Zhong, Tianquan Yang, Yong Han, Yongjun Xiang, Bin Yuan, Ruxuan Zhou, Min Chen, Hangzhou Wang

**Affiliations:** ^1^ Department of Neurosurgery, Children’s Hospital of Soochow University, Suzhou, Jiangsu, China; ^2^ Department of Pediatric Hematology and Oncology, Children’s Hospital of Soochow University, Suzhou, Jiangsu, China; ^3^ Institute of Pediatric Research, Children’s Hospital of Soochow University, Suzhou, Jiangsu, China

**Keywords:** glycemic variability, hemorrhagic stroke, MIMIC-IV database, prognosis, intensive care unit (ICU)

## Abstract

**Background:**

Elevated glycemic variability (GV) is commonly observed in intensive care unit (ICU) patients and has been associated with clinical outcomes. However, the relationship between GV and prognosis in ICU patients with hemorrhagic stroke (HS) remains unclear. This study aims to investigate the association between GV and short- and long-term all-cause mortality.

**Methods:**

Clinical data for hemorrhagic stroke (HS) patients were obtained from the MIMIC-IV 3.1 database. GV was quantified using the coefficient of variation (CV), calculated as the ratio of the standard deviation to the mean blood glucose level. The association between GV and clinical outcomes was analyzed using Cox proportional hazards regression models. Additionally, restricted cubic spline (RCS) curves were employed to examine the nonlinear relationship between GV and short- and long-term all-cause mortality.

**Results:**

A total of 2,240 ICU patients with HS were included in this study. In fully adjusted models, RCS analyses revealed a U-shaped association between the CV and both short- and long-term all-cause mortality (P for nonlinearity < 0.001 for all outcomes). Two-piecewise Cox regression models were subsequently applied to identify CV thresholds. The thresholds for all-cause mortality in ICU, during hospitalization, and at 30, 90, and 180 days were determined to be 0.14, 0.16, 0.155, 0.14, and 0.14, respectively. These findings were consistent in sensitivity and subgroup analyses.

**Conclusions:**

In HS patients, higher GV is associated with an increased risk of both short- and long-term all-cause mortality. Our findings suggest that stabilizing GV may improve the prognosis of HS patients.

## Background

Cerebrovascular disease (CVD) is the second leading cause of death worldwide, surpassed only by cardiovascular disease (1–3). Stroke, a major component of CVD, has been identified by the World Health Organization as the primary cause of long-term disability globally (4, 5). Although hemorrhagic stroke (HS) accounts for only 10–20% of all stroke cases, it is responsible for nearly half of all stroke-related deaths (6, 7). With an aging global population, the burden of stroke continues to rise, with HS patients in intensive care units (ICU) facing an elevated mortality risk (8). Consequently, identifying prognostic markers for predicting adverse outcomes in HS patients is essential. Historically, assessment tools such as the NIH Stroke Scale and the Canadian Neurological Scale have been widely utilized (9). Despite their utility, these scales are limited by their complexity, time requirements, and the need for specialized training.

Recently, glycemic variability (GV), a parameter of glycemic control, has emerged as a potential factor influencing the progression of cardiovascular and cerebrovascular conditions (10–13). GV reflects fluctuations in blood glucose levels relative to the mean and represents a key pattern of glycemic dysregulation observed in critically ill patients. Compared to persistent hyperglycemia, pronounced glycemic variability has been demonstrated to exacerbate endothelial dysfunction and trigger oxidative stress, potentially leading to more severe cerebrovascular damage (14–16). Moreover, both hyperglycemia and hypoglycemia were recognized as significant factors influencing stroke prognosis (16). Despite this, the impact of glycemic variability on HS patients has been understudied and remains a topic of debate in clinical practice (17, 18).

To address this gap, the present study examined the association between glycemic variability and short-term and long-term all-cause mortality in HS patients. The findings aimed to support clinicians in identifying high-risk individuals, facilitating closer monitoring and timely therapeutic interventions.

## Method

### Data source

The present study conducted a retrospective analysis using the Medical Information Mart for Intensive Care IV (MIMIC-IV) 3.1 database. MIMIC-IV is a publicly accessible dataset that contains comprehensive medical records of patients admitted to intensive care units at Beth Israel Deaconess Medical Center in Boston, Massachusetts, between 2008 and 2019 (19). The database provides detailed clinical information, including patient demographics, laboratory results, medication records, vital signs, surgical procedures, diagnoses, and treatment outcomes. Data extraction was performed by Yuchen Liu, who completed the required training and certification to access and utilize the database (Record ID: 13,284,033). Stringent procedures were followed during data extraction to ensure accuracy and consistency.

### Study population

The analysis included patients diagnosed with non-traumatic subarachnoid hemorrhage and non-traumatic intracerebral hemorrhage, as defined by the International Classification of Diseases, 9th and 10th editions (ICD-9 and ICD-10). The exclusion criteria were as follows: (1) patients not admitted to the ICU; (2) patients with fewer than three blood glucose measurements to ensure the accuracy and reliability of the GV assessment; (3) patients with a hospital stay of less than 24 hours or who died within 24 hours of admission; (4) individuals diagnosed with malignancies. We only included data from their first admission for patients with multiple admissions.

### Clinical data collection

(1) Demographics: gender, age, and ethnicity; (2) Clinical severity: Glasgow Coma Scale (GCS), Sequential Organ Failure Assessment (SOFA) score, Simplified Acute Physiology Score II, Acute Physiology Score III, and Oxford Acute Severity of Illness Score.(3) Comorbidities: hypertension, diabetes, intraventricular hemorrhage (IVH), chronic obstructive pulmonary disease, myocardial infarction, congestive heart failure, peripheral vascular disease, renal failure, hepatic disorders, sepsis, and Charlson Comorbidity Index;(4) Vital signs: heart rate, systolic blood pressure (SBP), diastolic blood pressure, mean blood pressure, Temperature, and percutaneous oxygen saturation; (5) Laboratory results: hemoglobin, white blood cell count (WBC), hematocrit, sodium, potassium, blood platelet count, serum creatinine (Scr), blood urea nitrogen, prothrombin time (PT), activated partial thromboplastin time, international normalized ratio, and blood glucose; (6) Treatment: mechanical ventilation, anti-diabetes therapy and use of vasopressor; (7)Prognosis: survival information, the length of hospital and ICU stay.

### Glycemic variability

The GV was based on real-time blood glucose records obtained during the hospital stay, with measurements taken prior to the occurrence of patient outcomes. Given that this study is a retrospective real-world analysis, the frequency of blood glucose testing was not standardized. Therefore, the coefficient of variation (CV) was selected to describe GV, calculated as the ratio of the standard deviation to the mean of all multiple measurements. This is consistent with previous literature (11, 20, 21).

### Outcomes

The primary outcomes of the present study were short-term all-cause mortality (ICU stay, hospitalization, and 30 days), while the secondary outcomes were long-term all-cause mortality (90 days and 180 days).

### Statistical analysis

The proportion of missing variables was presented in **Supplementary Figure S1**. Multiple imputation was used to address missing data. Subjects were stratified into tertiles based on their CV values (T1-T3). Quantitative variables are presented as mean ± standard deviation or median and interquartile range (IQR), depending on the data distribution, while qualitative variables are expressed as counts and proportions. The t-test or analysis of variance was used for continuous variables with a normal distribution, whereas the Mann-Whitney U test or Kruskal-Wallis test was employed for non-normally distributed variables. Pearson’s chi-squared test was used to compare categorical variables. The incidence of all-cause mortality was assessed for each tertile during the observation period.

First, Cox proportional hazard models were employed to quantify the association between CV and the study endpoints, generating hazard ratios and 95% confidence intervals. In this analysis, CV was examined both as a continuous and categorical variable. Covariates for the adjusted model were selected based on their associations with the outcomes of interest or a change in the effect estimate exceeding 10% (**Supplementary Table S1**). Spearman’s rank correlation test was performed to assess multicollinearity in the Cox regression analysis, and the square root of the variance inflation factor was calculated. Furthermore, a restricted cubic spline (RCS) with four knots (at the 5th, 35th, 65th, and 95th percentiles) was used to flexibly represent the relationship between CV and the outcomes, exploring potential nonlinear associations. Based on the shape of the exposure-effect relationship, CV values were divided into three groups, with the lowest level serving as the reference group. If the correlation was nonlinear, a recursive algorithm was used to determine the inflection point between CV and the study outcomes. A two-stage Cox proportional hazards model was applied on both sides of the inflection point to further investigate the relationship between CV and mortality at different time points. Stratified and interaction analyses were conducted to examine the effects of gender, age (under 65 years or 65 years and above), and the presence of hypertension and diabetes. The likelihood ratio test was used to detect interactions.

R software (version 4.2.1; R Foundation for Statistical Computing; https://www.R--project.org), the R survey package (version 4.1-­1), and Free Statistics software (version 1.9.2; Beijing Free Clinical Medical Technology Co., Ltd.) were used for analyses. A two-sided p-value < 0.05 was considered statistically significant.

## Results

### Baseline characteristics

**Figure 1:**
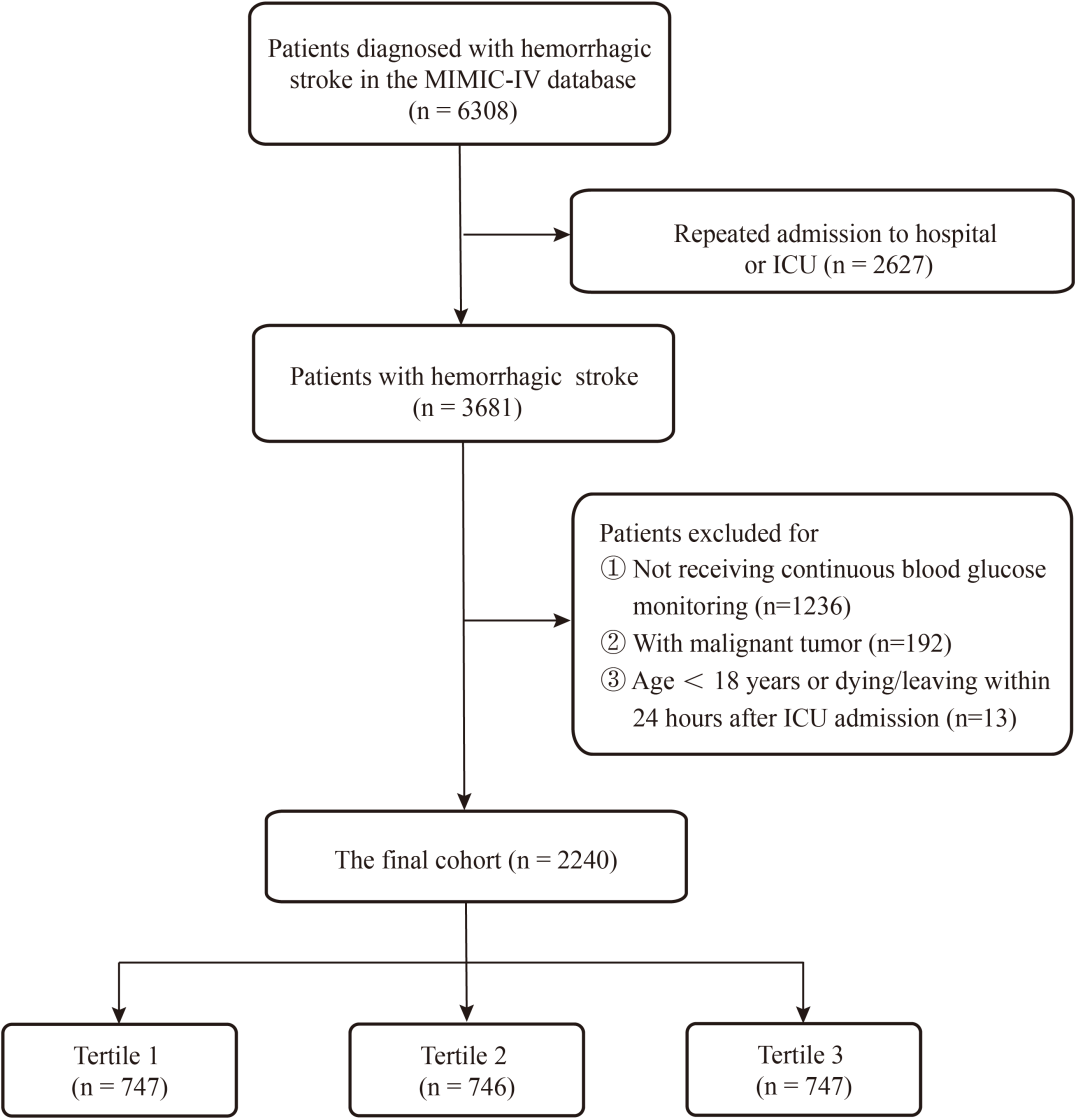
Flowchart for study participants enrolling.

Based on the inclusion criteria, a total of 6,308 patients were initially identified from the MIMIC-IV database. After applying the exclusion criteria, 4,068 patients were excluded, leaving 2,240 subjects for the final analysis (**Figure 1**). Among these, 1,142 (51%) were male, with a median age of 65 years (IQR: 53–77) and a median CV of 0.16 (IQR: 0.11–0.22). The median total blood glucose measurement count was 8 (IQR: 4–16) times during the ICU stay. Participants were stratified into three groups based on tertiles of CV: T1 (median: 0.09, IQR: 0.07–0.11), T2 (median: 0.16, IQR: 0.14–0.17), and T3 (median: 0.27, IQR: 0.22–0.35). Detailed baseline characteristics are presented in **Table 1**.

**Table 1:** Clinical characteristics of the study participants.

Variables	Total (*n* = 2240)	T1 (*n* = 747)	T2 (*n* = 746)	T3 (*n* = 747)	*P* value
CV	0.16 (0.11, 0.22)	0.09 (0.07, 0.11)	0.16 (0.14, 0.17)	0.27 (0.22, 0.35)	< 0.001
Blood glucose times	8 (4, 16)	5 (4, 10)	10 (6, 19)	9 (5, 20)	< 0.001
Demographic variables					
Age, years	65 (53, 77)	65 (53, 77)	64 (53, 76)	65 (54, 76)	0.541
Male	1142 (51.0)	380 (50.9)	377 (50.5)	385 (51.5)	0.925
Race (White)	1205 (53.8)	426 (57)	396 (53.1)	383 (51.3)	0.074
Clinical severity					
GCS	11 (7, 14)	13 (9, 14)	10 (7, 13)	9 (6, 14)	< 0.001
SOFA	5 (3, 7)	4 (2, 5)	5 (3, 7)	6 (4, 8)	< 0.001
SAPS-II	32 (24, 40)	29 (22, 37)	32 (25, 39)	35 (27, 44)	< 0.001
OASIS	34 (27, 40.2)	31 (25, 38)	35 (28, 40)	36 (29, 43)	< 0.001
APSIII	41 (29, 58.2)	35 (26, 48)	42 (30, 56)	50 (34, 73)	< 0.001
Comorbidities					
Hypertension	509 (22.7)	159 (21.3)	190 (25.5)	160 (21.4)	0.09
Diabetes	491 (21.9)	99 (13.3)	120 (16.1)	272 (36.4)	< 0.001
IVH	306 (13.7)	91 (12.2)	109 (14.6)	106 (14.2)	0.344
COPD	296 (13.2)	93 (12.4)	99 (13.3)	104 (13.9)	0.701
Myocardial infarct	189 (8.4)	46 (6.2)	61 (8.2)	82 (11)	0.003
Congestive heart failure	282 (12.6)	82 (11)	73 (9.8)	127 (17)	< 0.001
PVD	164 (7.3)	43 (5.8)	63 (8.4)	58 (7.8)	0.116
Renal failure	240 (10.7)	59 (7.9)	61 (8.2)	120 (16.1)	< 0.001
Hepatic disorders	48 (2.1)	11 (1.5)	14 (1.9)	23 (3.1)	0.083
Sepsis	1131 (50.5)	269 (36)	416 (55.8)	446 (59.7)	< 0.001
CCI	6 (4, 7)	5 (4, 7)	5 (4, 7)	6 (4, 8)	< 0.001
Vital signs					
SBP, mmHg	128.5 ± 13.6	128.9 ± 12.8	129.1 ± 13.4	127.4 ± 14.5	0.029
DBP, mmHg	67.2 ± 10.9	67.9 ± 10.4	67.3 ± 10.6	66.4 ± 11.7	0.022
MBP, mmHg	85.0 ± 10.0	85.5 ± 9.5	85.6 ± 9.8	84.0 ± 10.5	0.003
Heart rate, beats/min	80.3 ± 13.7	78.2 ± 12.7	80.1 ± 13.6	82.6 ± 14.3	< 0.001
Temperature, °C	37.0 ± 0.5	37.1 ± 0.4	37.1 ± 0.4	37.0 ± 0.6	0.007
SpO2, %	97.3 ± 1.9	97.1 ± 1.8	97.5 ± 1.8	97.4 ± 2.0	< 0.001
Laboratory Parameters					
Hb, g/L	11.8 ± 2.1	12.1 ± 1.9	11.8 ± 2.1	11.6 ± 2.2	< 0.001
WBC, 10^9^/L	9.8 (7.7, 12.4)	9.5 (7.7, 11.9)	9.7 (7.7, 12.4)	10.1 (7.8, 13.0)	0.02
Hematocrit, vol%	35.5 ± 6.0	36.3 ± 5.4	35.5 ± 5.9	34.8 ± 6.5	< 0.001
Sodium, mmol/L	137.8 ± 4.5	138.0 ± 4.0	137.9 ± 4.0	137.4 ± 5.2	0.021
Potassium, mmol/L	3.7 ± 0.5	3.7 ± 0.4	3.7 ± 0.5	3.7 ± 0.5	0.032
Platelets, 10^9^/L	198 (158, 246)	202 (166, 246.5)	197 (158, 244)	197 (149.5, 246.0)	0.093
Serum creatinine, mg/dL	0.8 (0.6, 1.0)	0.8 (0.6, 1.0)	0.8 (0.6, 1.0)	0.8 (0.6, 1.1)	< 0.001
Urea nitrogen, mg/dL	14 (10, 19)	13 (10, 18)	13 (10, 18)	15 (11, 22)	< 0.001
PT, s	12.1 (11.3, 13.1)	12.1 (11.4, 13.0)	12 (11.3, 12.9)	12.3 (11.4, 13.3)	0.013
APTT, s	26.8 (24.3, 29.4)	27.2 (24.8, 29.4)	26.6 (24.3, 29.0)	26.6 (23.9, 29.5)	0.027
INR	1.1 ± 0.2	1.1 ± 0.2	1.1 ± 0.3	1.2 ± 0.2	0.12
Treatment					
Mechanical ventilation	1282 (57.2)	328 (43.9)	457 (61.3)	497 (66.5)	< 0.001
Vasopressor	831 (37.1)	188 (25.2)	275 (36.9)	368 (49.3)	< 0.001
Anti-diabetes therapy	1490 (66.8)	430 (57.9)	506 (67.9)	554 (74.7)	< 0.001
Outcomes					
short-term mortality					
ICU mortality	338 (15.1)	69 (9.2)	91 (12.2)	178 (23.8)	< 0.001
In-hospital mortality	439 (19.6)	104 (13.9)	121 (16.2)	214 (28.6)	< 0.001
30-day mortality	508 (22.7)	127 (17)	146 (19.6)	235 (31.5)	< 0.001
long-term mortality					
90-day mortality	603 (26.9)	148 (19.8)	172 (23.1)	283 (37.9)	< 0.001
180-day mortality	658 (29.4)	165 (22.1)	191 (25.6)	302 (40.4)	< 0.001

CV, coefficient of variation; GCS, Glasgow Coma Scale; SOFA, Sequential Organ Failure Assessment; SAPS-II, Simplified Acute Physiology Score II; APS III, Acute Physiology Score III; OASIS, Oxford Acute Severity of Illness Score; IVH, intraventricular hemorrhage; COPD, chronic obstructive pulmonary disease; PVD, peripheral vascular disease; CCI, Charlson Comorbidity Index; SBP, systolic blood pressure; DBP, diastolic blood pressure; MBP, mean blood pressure; Hb, hemoglobin; WBC, white blood cell count; PT, prothrombin time; APTT, activated partial thromboplastin time; INR, international normalized ratio.

### Association of CV and all-cause mortality

**Figure 2:**
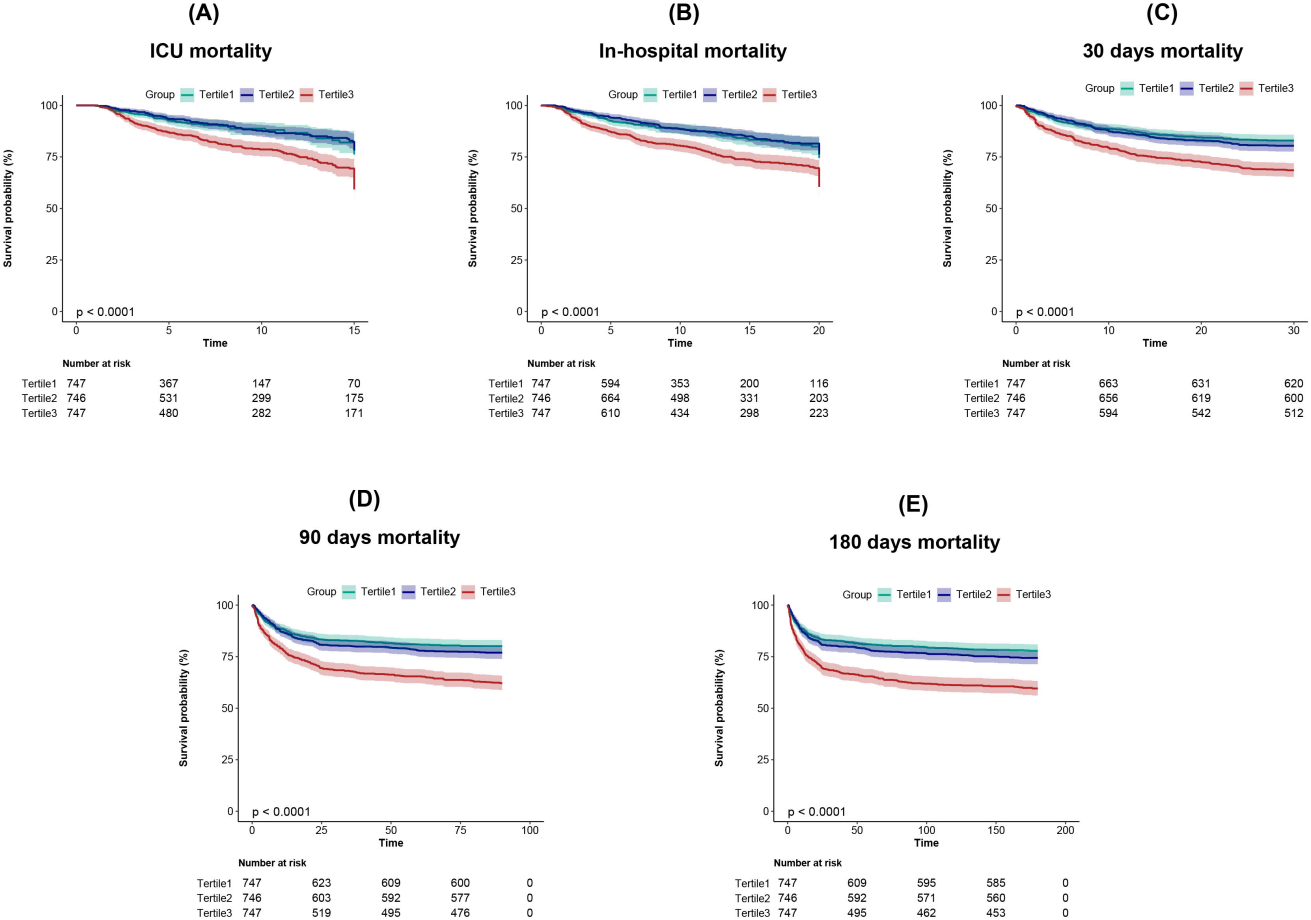
Kaplan-Meier survival analysis curves for **(A)** ICU, **(B)** In-hospital, **(C)** 30 days, **(D)** 90 days, and **(E)** 180 days all-cause mortality.

The Kaplan-Meier curves demonstrate that the short-term and long-term cumulative survival rates in patients with hemorrhagic stroke decrease significantly and progressively in the order of T1 > T2 > T3 (**Figure 2**). Cox regression analysis was used to evaluate the relationship between CV and all-cause mortality at different time intervals (ICU stay, hospitalization, 30 days, 90 days, and 180 days). The results indicated that when analyzed as a continuous variable, CV was a significant predictor of mortality across all time intervals in both the unadjusted Model 1 and the partially adjusted Model 2. However, statistical significance was not observed in the fully adjusted Model 3. This model adjusted for age, sex, ethnicity, SOFA score, GCS, hypertension, diabetes, IVH, myocardial infarction, SBP, WBC, blood platelet count, sodium, potassium, Scr, PT, mechanical ventilation, anti-diabetes therapy, and vasopressor use.

Given this paradoxical phenomenon, T2 was used as the reference group in the Cox proportional hazards model. Interestingly, when CV was treated as a categorical variable, patients in the lowest and highest CV tertiles demonstrated significantly increased risks of short-term and long-term all-cause mortality in the fully adjusted Model 3 (**Table 2**).

**Table 2:** Cox proportional hazard ratios for short- and long-term all-cause mortality.

Variable	Model 1		Model 2		Model 3	
	HR (95%CI)	*P* value	HR (95%CI)	*P* value	HR (95%CI)	*P* value
ICU mortality						
CV	4.1 (2.56~6.58)	<0.001	4.72 (2.87~7.78)	<0.001	1.51 (0.79~2.89)	0.215
Tertile						
T1 (*n*=747)	1.1 (0.8~1.5)	0.567	1.11 (0.81~1.52)	0.507	1.48 (1.13~1.94)	0.005
T2 (*n*=746)	Reference		Reference		Reference	
T3 (*n*=747)	2.02 (1.57~2.61)	<0.001	1.95 (1.52~2.51)	<0.001	1.3 (1.02~1.65)	0.031
In-hospital mortality						
CV	3.23 (2.08~5.01)	<0.001	3.37 (2.15~5.3)	<0.001	2.41 (1.2~4.84)	0.013
Tertile						
T1 (*n*=747)	1.06 (0.81~1.38)	0.675	1.06 (0.81~1.38)	0.67	1.55 (1.12~2.14)	0.008
T2 (*n*=746)	Reference		Reference		Reference	
T3 (*n*=747)	1.84 (1.47~2.3)	<0.001	1.78 (1.42~2.23)	<0.001	1.37 (1.05~1.8)	0.022
30 days mortality						
CV	3.98 (2.69~5.9)	<0.001	4.07 (2.72~6.07)	<0.001	1.81 (0.98~3.34)	0.058
Tertile						
T1 (*n*=747)	0.87 (0.68~1.1)	0.239	0.87 (0.69~1.11)	0.268	1.33 (1.04~1.7)	0.022
T2 (*n*=746)	Reference		Reference		Reference	
T3 (*n*=747)	1.75 (1.43~2.16)	<0.001	1.71 (1.39~2.11)	<0.001	1.28 (1.02~1.6)	0.03
90 days mortality						
CV	4.07 (2.86~5.8)	<0.001	4.02 (2.81~5.76)	<0.001	1.75 (1.02~3)	0.041
Tertile						
T1 (*n*=747)	0.85 (0.68~1.06)	0.157	0.85 (0.69~1.06)	0.158	1.31 (1.04~1.64)	0.019
T2 (*n*=746)	Reference		Reference		Reference	
T3 (*n*=747)	1.82 (1.51~2.2)	<0.001	1.78 (1.47~2.15)	<0.001	1.3 (1.06~1.6)	0.011
180 days mortality						
CV	3.91 (2.77~5.51)	<0.001	3.79 (2.68~5.37)	<0.001	1.62 (0.97~2.72)	0.067
Tertile						
T1 (*n*=747)	0.85 (0.69~1.05)	0.138	0.85 (0.69~1.05)	0.13	1.29 (1.04~1.6)	0.018
T2 (*n*=746)	Reference		Reference		Reference	
T3 (*n*=747)	1.76 (1.47~2.12)	<0.001	1.72 (1.44~2.06)	<0.001	1.27 (1.04~1.54)	0.017

Model 1: unadjusted; Model 2: adjusted age, sex, ethnicity; Model 3: adjusted for age, sex, ethnicity, SOFA score, GCS, hypertension, diabetes, IVH, myocardial infarction, SBP, WBC, blood platelet count, sodium, potassium, Scr, PT, mechanical ventilation, anti-diabetes therapy, and vasopressor use.

### U-shaped impact of CV on mortality outcomes

**Figure 3:**
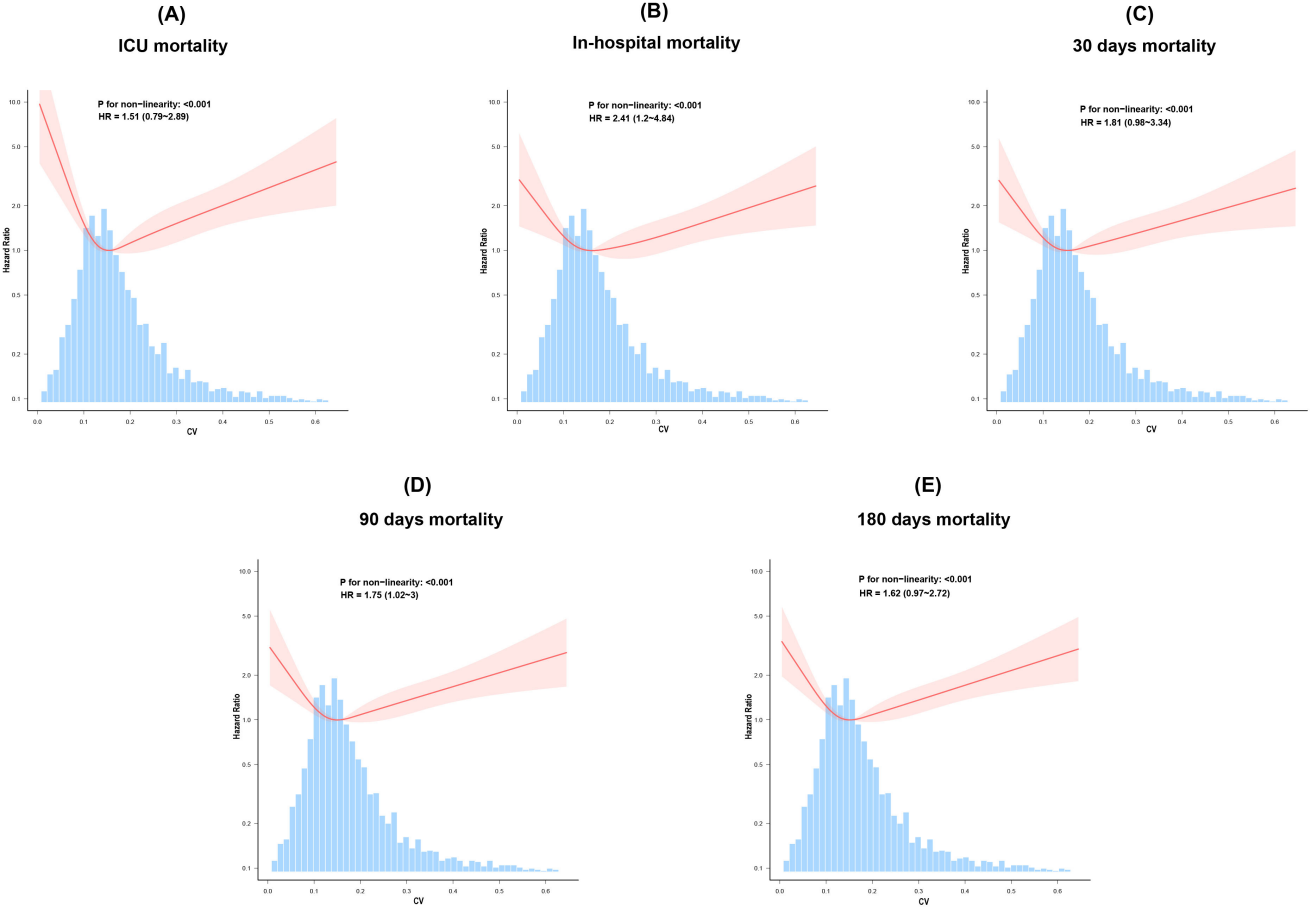
The restricted cubic spline curves, adjusted for age, sex, ethnicity, SOFA score, GCS, hypertension, diabetes, IVH, myocardial infarction, SBP, WBC, platelet count, sodium, potassium, Scr, PT, mechanical ventilation, anti-diabetes therapy, and vasopressor use, illustrate the relationship between glycemic variability and all-cause mortality **(A)** ICU, **(B)** In-hospital,**(C)** 30 days, **(D)** 90 days, and **(E)** 180 days.

The RCS was used to illustrate the relationship between CV and all-cause mortality flexibly. The analysis revealed a nonlinear increase in mortality risk during ICU stay, during hospitalization, and at 30, 90, and 180 days as CV rose after adjusting for confounders in the fully adjusted Model 3 (P for non-linearity < 0.001 for study outcomes) (**Figure 3**).

Two-piecewise Cox regression models were further used to determine the threshold for CV. For all-cause mortality outcomes at ICU discharge, during hospitalization, and at 30, 90, and 180 days, the CV thresholds are 0.14, 0.16, 0.155, 0.14, and 0.14, respectively. As shown in **Table 3**, when CV was below 0.155, each unit increase in CV is associated with a 99.7% reduction in the risk of 30-day mortality (HR: 0.003; 95% CI: 0–0.438; P = 0.0223). Conversely, when CV exceeds this threshold, each unit increase in CV was associated with a 9.887-fold higher risk of 30-day mortality (HR: 10.887; 95% CI: 3.253–36.437; P < 0.001). The U-shaped associations between CV and other study outcomes are presented in **Table 3**.

**Table 3:** Threshold effect analysis of the relationship between CV and all-cause mortality.

CV	HR (95%CI)	P value
ICU mortality		
<0.14	0 (0,0.001)	0.024
≥0.14	18.839 (6.7,52.973)	< 0.001
Likelihood Ratio test		< 0.001
In-hospital mortality		
<0.16	0.016 (0.001,0.289)	0.007
≥0.16	14.742 (4.015,54.125)	< 0.001
Likelihood Ratio test		0.002
30 days mortality		
<0.155	0.003 (0,0.438)	0.0223
≥0.155	10.887 (3.253,36.437)	< 0.001
Likelihood Ratio test		<0.001
90 days mortality		
<0.14	0 (0,0.053)	0.0025
≥0.14	9.963 (3.54,28.035)	< 0.001
Likelihood Ratio test		<0.001
180 days mortality		
<0.14	0 (0,0.006)	< 0.001
≥0.14	9.847 (3.671,26.409)	< 0.001
Likelihood Ratio test		<0.001

CV, coefficient of variation.

### Subgroup analysis

**Figure 4:**
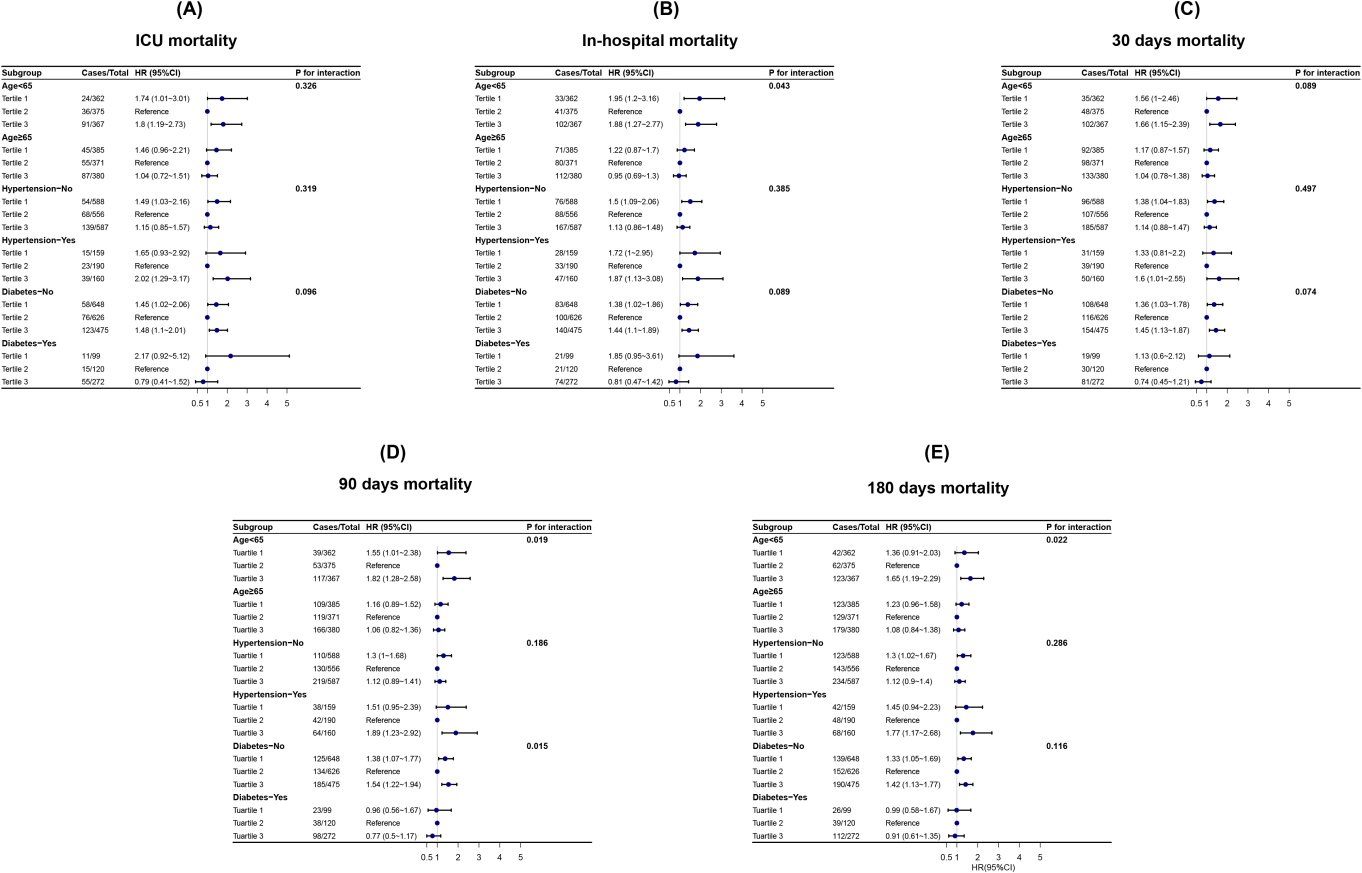
Subgroup analysis between CV and all-cause mortality.

To further validate the association between CV and short-term mortality in hemorrhagic stroke patients, as well as the significant interactions between CV and patient age, hypertension, and diabetes regarding short-term mortality, sensitivity analyses were conducted using Cox regression. After full adjustment, U-shaped relationships were observed between CV and short-term and long-term mortality in patients under 65 years of age, patients without diabetes, and patients with hypertension (**Figure 4**).

Moreover, no significant interactions were identified between CV and age, hypertension, or diabetes concerning short-term mortality in hemorrhagic stroke patients. However, subgroup analysis revealed a significant interaction between CV and age (< 65 years vs. ≥ 65 years) (In-hospital: P for interaction = 0.043; 90-day: P for interaction = 0.019; 180-day: P for interaction = 0.022), indicating a stronger association between CV and long-term mortality in patients under 65 years of age. Additionally, for 90-day all-cause mortality after ICU admission, subgroup analysis reveals a significant interaction between CV and diabetes status (P for interaction = 0.015), highlighting a stronger association between CV and 90-day mortality in patients without diabetes. These findings suggest that CV may serve as a prognostic indicator for short-term mortality in patients with hemorrhagic stroke and long-term survival in patients under 65 years of age and without diabetes (**Figure 4**).

### Sensitivity analysis

**Figure 5:**
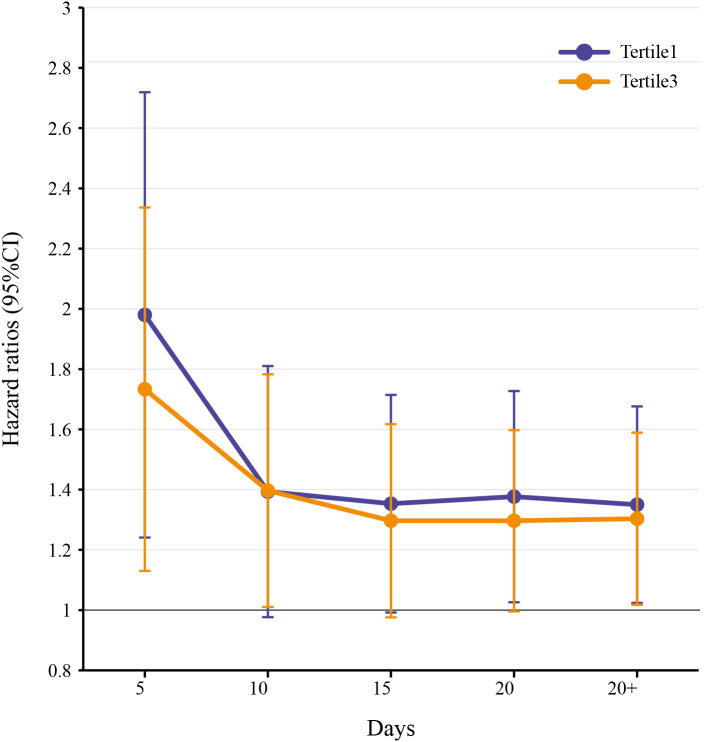
Analysis of the correlation between in-hospital mortality and CV in HS patients in 4 different periods.

The sensitivity analysis strategy involved selecting four different time points (Day 5, Day 10, Day 15, and Day 20 after admission) to assess the association between CV and all-cause mortality in patients with hemorrhagic stroke using Cox regression analysis. The results showed that, compared to the second CV tertile, the first CV tertile was associated with an increased risk of mortality at Day 5 [HR (95% CI) = 1.89 (1.29–2.76), P = 0.001], Day 15 [HR (95% CI) = 1.32 (1.01–1.73), P = 0.046], Day 20 [HR (95% CI) = 1.35 (1.04–1.74), P = 0.024], and overall [HR (95% CI) = 1.32 (1.04–1.69), P = 0.024]. Similarly, the third CV tertile was associated with an increased risk of mortality at Day 5 [HR (95% CI) = 1.66 (1.17–2.37), P = 0.005], Day 10 [HR (95% CI) = 1.36 (1.03–1.80), P = 0.028], Day 20 [HR (95% CI) = 1.27 (1.01–1.61), P = 0.045], and overall [HR (95% CI) = 1.28 (1.03–1.60), P = 0.028] (**Figure 5**).

## Discussion

Based on a large ICU database, we evaluated the impact of GV on short-term and long-term all-cause mortality in patients with HS. The results indicated that mortality risk increased with higher CV levels. However, after full adjustment, both short-term and long-term mortality exhibited a U-shaped trend across CV levels, reaching the lowest point at a CV range of approximately 0.14–0.16. Multivariable-adjusted Cox analysis further confirmed this U-shaped association. In subgroup analyses stratified by age, hypertension, and diabetes, the U-shaped relationship between CV and mortality persisted in patients under 65 years of age, patients without diabetes, and patients with hypertension. Notably, the association between CV and long-term mortality was more pronounced in HS patients under 65 years of age. In summary, this study demonstrated a U-shaped relationship between GV and short-term and long-term mortality in patients with HS. Accordingly, GV emerges as a viable marker for stratifying and managing stroke patients during long-term follow-up care.

The global rise in obesity rates has been directly linked to an increased incidence of CVD (22). Obesity contributes to stroke through multiple mechanisms, including glucose metabolism disorders, chronic low-grade inflammation, endothelial dysfunction, lipid abnormalities, hypertension, and coagulation disorders. Among these factors, glucose metabolism plays a critical role in maintaining the physiological integrity of the cardiovascular and cerebrovascular systems (23). Disruption of this metabolic balance serves as a pivotal driver for the onset and progression of CVD. Numerous studies have demonstrated that lipid alterations associated with fasting blood glucose levels, such as the triglyceride-glucose index and the stress hyperglycemia ratio, are strongly correlated with the occurrence of CVD, impaired consciousness, and poor prognosis (9, 10, 12, 13, 20). These findings highlight the significant impact of glycemic instability on exacerbating adverse outcomes in CVD.

Recent consensus statements on the interpretation of continuous glucose monitoring data in patients with diabetes have suggested that the CV should be used as the primary metric for assessing GV (24, 25). Previous studies have confirmed that a high CV is associated with an increased risk of long-term cognitive decline in the general population (26). Furthermore, several studies have demonstrated that elevated CV is linked to a higher risk of major adverse cardiovascular events and all-cause mortality in patients with cardiovascular diseases (10, 12, 13, 20). Given the prevalence of glycemic abnormalities among ICU patients, Ma et al. identified CV as a critical factor influencing all-cause mortality in critically ill individuals (27). Recently, Linrui et al. found that higher CV was significantly associated with lower GCS scores and an increased risk of in-hospital mortality in patients with traumatic brain injury (11).

Additionally, recent studies have demonstrated a strong association between CV and clinical outcomes in patients with acute stroke. Higher CV was typically linked to poorer neurological recovery and functional outcomes. One study reported that greater CV, represented by higher mean absolute glucose levels, was associated with a reduced likelihood of neurological improvement during hospitalization, even after adjusting for potential confounders (28). Another study found that hyperglycemia and elevated glucose standard deviation in patients with acute ischemic stroke correlated with worse functional status at discharge (29). Beyond functional outcomes, CV has also been implicated in mortality risk. In patients with severe acute stroke, glucose standard deviation and fluctuation amplitude were identified as independent predictors of mortality, suggesting that greater glucose fluctuations may indicate worse disease prognosis (30). Hyperglycemia in the acute phase of stroke may exacerbate brain injury and is associated with unfavorable clinical outcomes. Evidence indicated that elevated blood glucose levels were linked to worse outcomes in patients with non-lacunar stroke but not in those with lacunar stroke (31). Additionally, in patients with acute ischemic stroke undergoing intravenous thrombolysis, hyperglycemia significantly increases the risk of symptomatic intracranial hemorrhage and poor outcomes at 90 days (32).

However, research investigating the impact of CV on the prognosis of patients with HS remains limited. The study by Huang et al. on the association between CV and stroke prognosis included only patients with intracerebral hemorrhage (29). However, HS comprises both intracerebral hemorrhage and subarachnoid hemorrhage. Furthermore, their research focused solely on in-hospital outcomes. To complement these findings, our study revealed a U-shaped association between CV and both short- and long-term mortality in patients with HS. The CV threshold range identified in this study (0.14–0.16) provides a practical reference for glucose management in HS patients. The results indicated that CV was negatively correlated with mortality when below the inflection point, while the risk significantly increased above this point. Overly stringent glycemic control may lead to iatrogenic hypoglycemia, which can exacerbate cerebral ischemic injury. Severe glucose fluctuations can further intensify cerebrovascular damage through mechanisms such as oxidative stress and endothelial dysfunction.

Although this study offers new evidence supporting the prognostic value of CV, its clinical translation requires careful consideration. A key point of ongoing debate is whether increased CV represents a consequence rather than a cause of metabolic dysregulation in critical illness (33, 34). Therefore, CV-targeted interventions should prioritize the correction of underlying pathophysiological disturbances, such as infection control and stress hormone regulation, rather than relying solely on insulin-mediated stabilization. Additionally, future interventional trials must employ more refined designs to delineate the independent effects of CV from mean glucose levels and incorporate novel antidiabetic agents to overcome current therapeutic limitations.

Subgroup analysis in this study revealed that elevated CV was more likely to significantly increase the risk of adverse outcomes in HS patients who were under 65 years of age, hypertensive, or without diabetes. In younger patients, the negative impact of abnormal CV on prognosis aligns with previous findings (35). Moreover, the observation that antioxidant stress markers have been found to more effectively reduce the risk of adverse outcomes in non-hypertensive individuals may partially explain why hypertensive patients tend to have worse outcomes (36). Interestingly, CV demonstrated greater prognostic value in populations without diabetes. We speculate that this may be attributed to the fact that diabetes patients typically receive more frequent blood glucose monitoring and timely interventions to address glycemic fluctuations.

Our study demonstrates that CV is a highly effective clinical marker for the early identification of HS patients at risk of adverse outcomes. Unlike traditional predictors such as glycated hemoglobin or triglyceride-glucose index, CV dynamically reflects changes in a patient’s condition, offering a more responsive assessment compared to static admission test results (37). The management of patients in the ICU represents a cornerstone of clinical practice. CV, as a readily obtainable parameter during ICU stays, offers clinicians a valuable tool for the early identification of high-risk patients. By leveraging CV, healthcare providers may reduce mortality rates and enhance overall patient outcomes.

It is important to acknowledge several limitations of this study. First, the reliance on the MIMIC-IV database poses challenges in establishing definitive causal relationships, highlighting the need for further prospective studies to confirm our findings. Second, despite extensive adjustment for potential confounding factors in this study, collider bias may still be present. Owing to the limitations of the MIMIC-IV database, detailed information on patients’ glycemic management strategies or dynamic inflammatory status could not be obtained. These factors may simultaneously influence both GV and mortality. Future prospective studies should incorporate more comprehensive physiological and therapeutic data to further validate these findings. Finally, the CV cannot fully capture the dynamic features of blood glucose fluctuations, including the frequency and direction of these variations. Therefore, it is suggested that future studies incorporate continuous glucose monitoring data to explore more comprehensive indicators of GV.

## Conclusion

In summary, a U-shaped association between GV and both short-term and long-term mortality was observed in patients with hemorrhagic stroke. Age below 65 years and the absence of diabetes may act as modifiers of the relationship between GV and prognosis in these patients. Further research is needed to confirm the causal relationship between GV and disease progression in patients with hemorrhagic stroke.

## Data Availability

The original contributions presented in the study are included in the article/**Supplementary Material**. Further inquiries can be directed to the corresponding author.

## Supplementary material

Supplementary Figure 1 Display of missing value ratios.
