# Supplementary figures and images for: U−shaped association between the glycemic variability and prognosis in hemorrhagic stroke patients: a retrospective cohort study from the MIMIC-IV database
Source: Front Endocrinol (Lausanne). 2025 Apr 3;16:1546164. doi: 10.3389/fendo.2025.1546164 (PMC12003122; doi:10.3389/fendo.2025.1546164)

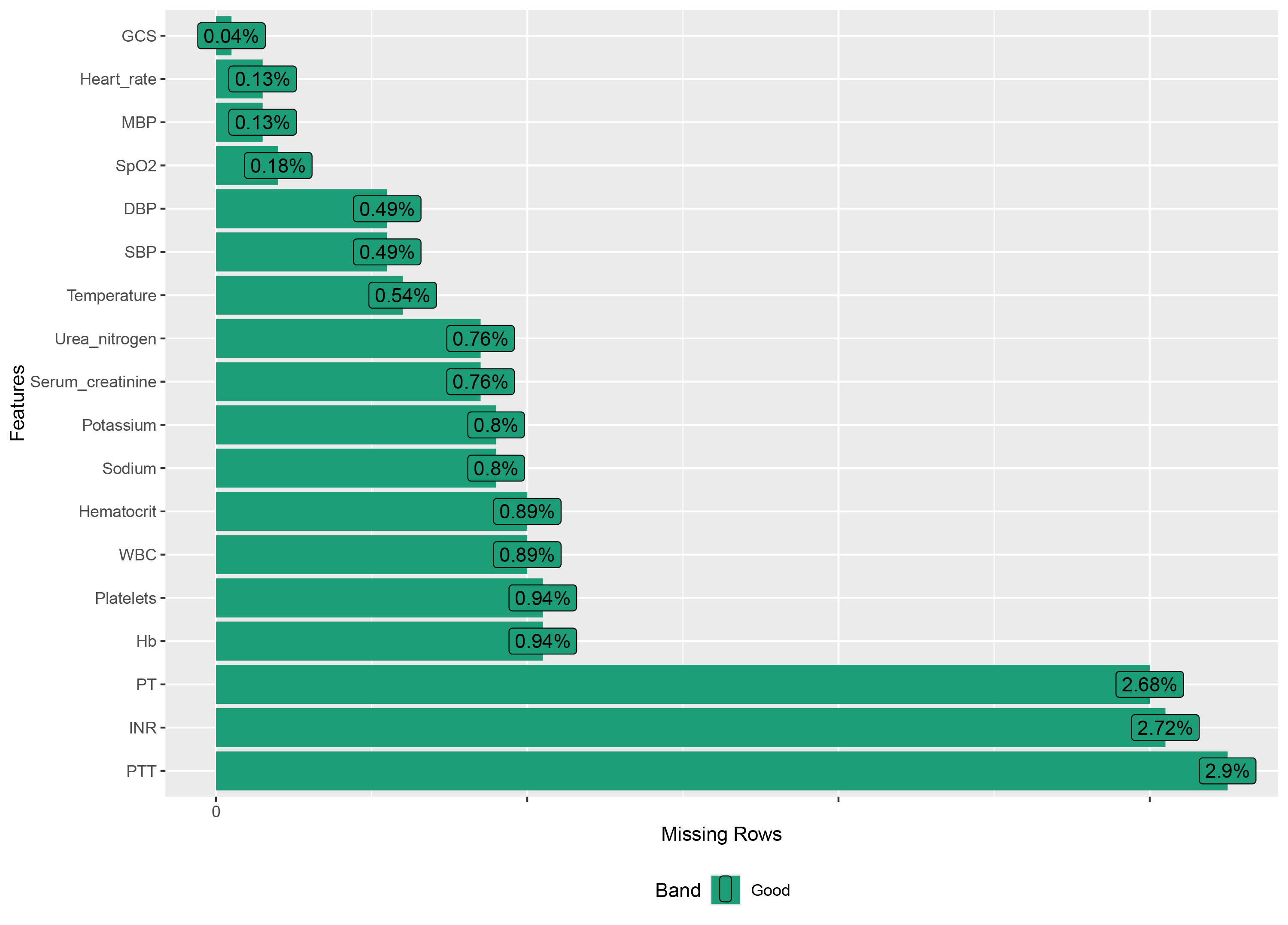

Supplement: Supplementary Figure 1 — Display of missing value ratios. [file Image1.tif]
