# Supplementary material for: U−shaped association between the glycemic variability and prognosis in hemorrhagic stroke patients: a retrospective cohort study from the MIMIC-IV database
Source: Front Endocrinol (Lausanne). 2025 Apr 3;16:1546164. doi: 10.3389/fendo.2025.1546164 (PMC12003122; doi:10.3389/fendo.2025.1546164)
Supplement: Supplementary file 2 [file Table1.docx]

| **Term1** | **coeff1** | **Change.percentage** | **Term2** | **coeff2** | **Change.percentage** | **VIF** | **colinearity** |
| --- | --- | --- | --- | --- | --- | --- | --- |
| Crude | 1.39 | Ref. | Full | 0.08 | Ref. | 1.363 | 0 |
| Age | 1.29 | -7.1 | Age | 0.07 | -8.1 | 2.221 | 0 |
| Male | 1.39 | 0 | Male | 0.08 | -2 | 1.269 | 0 |
| Ethnicty | 1.48 | 6.9 | Ethnicty | -0.07 | -188.3 | 1.135 | 0 |
| GCS | 1.16 | -16.3 | GCS | 0.31 | 305.1 | 2.965 | 0 |
| SOFA | 0.73 | -47.4 | SOFA | 0.06 | -18.3 | 3.738 | 0 |
| Hypertension | 1.39 | 0.1 | Hypertension | 0.15 | 89.1 | 1.382 | 0 |
| Diabetes_mellitus | 1.35 | -3 | Diabetes_mellitus | 0.09 | 20.3 | 1.434 | 0 |
| IVH | 1.4 | 0.6 | IVH | 0.06 | -22.1 | 1.174 | 0 |
| Myocardial_infarct | 1.38 | -0.8 | Myocardial_infarct | 0.08 | 10 | 1.189 | 0 |
| SBP | 1.38 | -0.5 | SBP | 0.05 | -40.7 | 3.432 | 0 |
| WBC | 1.38 | -0.9 | WBC | 0.06 | -25 | 1.126 | 0 |
| Platelets | 1.41 | 1.4 | Platelets | 0.04 | -47.8 | 1.292 | 0 |
| Sodium | 1.39 | -0.1 | Sodium | -0.01 | -118.4 | 1.387 | 0 |
| Potassium | 1.37 | -1.1 | Potassium | 0.1 | 33.2 | 1.466 | 0 |
| Serum_creatinine | 1.36 | -1.8 | Serum_creatinine | 0.04 | -45.3 | 2.442 | 0 |
| PT | 1.39 | 0.4 | PT | 0.09 | 13.2 | 2.803 | 0 |
| Mechanical_ventilation | 0.98 | -29.8 | Mechanical_ventilation | -0.12 | -250.9 | 1.073 | 0 |
| Vasopressor | 0.43 | -68.7 | Vasopressor | -0.06 | -174.2 | 1.043 | 0 |

**Supplementary Table 1** Selection of covariates.
